# Electrospun Polyacrylonitrile/Lignin/Poly(Ethylene Glycol)-Based Porous Activated Carbon Nanofiber for Removal of Nickel(II) Ion from Aqueous Solution

**DOI:** 10.3390/polym13203590

**Published:** 2021-10-19

**Authors:** Aiza Farhani Zakaria, Sazlinda Kamaruzaman, Norizah Abdul Rahman

**Affiliations:** 1 Department of Chemistry, Faculty of Science, Universiti Putra Malaysia, UPM, Serdang 43400, Selangor, Malaysia; aizafarhani97@gmail.com (A.F.Z.); sazlinda@upm.edu.my (S.K.); 2 Natural Medicines and Product Research Laboratory (NaturMeds), Institute of Bioscience (IBS), Universiti Putra Malaysia, UPM, Serdang 43400, Selangor, Malaysia; 3 Materials Processing and Technology Laboratory, Institute of Advanced Technology, Universiti Putra Malaysia, UPM, Serdang 43400, Selangor, Malaysia

**Keywords:** adsorption, carbon nanofiber, activated carbon nanofibers, nickel(II) ion, electrospinning, selective dissolution

## Abstract

The issue of heavy metal contamination has caused a great deal of concern among water quality experts today, as it contributes to water pollution. Activated carbon nanofibers (ACNFs) showed a significant ability in removing heavy metals from the wastewater. In this study, polyacrylonitrile (PAN) was blended and electrospun with an abundant and inexpensive biopolymer, lignin and a water soluble polymer, poly(ethylene glycol) (PEG), by using an electrospinning technique to form nanofibers. The electrospun nanofibers were then investigated as a precursor for the production of porous ACNFs to study the removal of nickel(II) ions by adsorption technique. PEG was added to act as a porogen and to create the porous structure of carbon nanofibers (CNFs). CNFs were prepared by thermal treatment of the electrospun nanofibers and followed by activation of CNFs by thermal and acid treatment on CNFs. Attenuated total reflectance Fourier transform infrared spectroscopy (ATR-FTIR) spectral analysis of the ACNFs showed a strong absorption peak of the C-O functional group, indicating the increase in the oxygenated compound. Field emission scanning electron microscopy (FESEM) images concluded that the ACNFs have more porous and compact fibers with a smaller fiber diameter of 263 ± 11 nm, while the CNFs are less compact and have slightly larger fiber diameter of 323 ± 6 nm. The adsorption study showed that the ACNFs possessed a much higher adsorption capacity of 18.09 mg/g compared with the CNFs, which the amount adsorbed was achieved only at 2.7 mg/g. The optimum adsorption conditions that gave the highest percentage of 60% for nickel(II) ions removal were 50 mg of adsorbent dosage, 100 ppm of nickel(II) solution, pH 3, and a contact time of 60 min. The study demonstrated that the fabrication of ACNFs from PAN/lignin/PEG electrospun nanofibers have potential as adsorbents for the removal of heavy metal contaminants.

## 1. Introduction

Untreated industrial effluents containing large amounts of heavy metals that are discharged into major water resources such as rivers and lakes by some manufacturing industries has brought a source of danger to abiotic and biotic ecosystems, especially to the health of living organisms such as humans, plants, and animals when exposed to long-term pollution [1,2]. Heavy metals such as lead, nickel, chromium, copper, and phosphorus are quite possibly the most genuine sorts of toxins and are hard to separate; surprisingly, limited amounts can represent an extraordinary danger to the climate and humans that can affect the capacity of the focal sensory system, leading to psychological issues [3,4,5,6,7,8]. However, the expansive use of nickel has been widely utilized as raw materials in the manufacturing industries, such as construction, car batteries, agricultural plantations, electroplating, fertilizers, and electronic equipment, have certainly created an issue with attested implications for ecosystem prosperities. Nickel is one of the chemical substances that are toxic, stable, and persistently carcinogenic towards the well-being of living organisms, the aquatic environment, and water body supply if left untreated [9]. It can form free progressives from diatomic molecules by twofold development action and generate superoxide anions that combine with protons and work with dismutation to design hydrogen peroxide and cause the main reason for pathophysiological changes triggered in living structures [10,11]. Therefore, the problem of nickel contamination in industrial wastewater is the main concern of researchers globally, which led to the development of various methods such as chemical precipitation, ion exchange, membrane filtration, solvent extraction, coagulation, and adsorption [12]. However, among the listed methods, adsorption technique is the most efficient because it is less expensive, easy to regenerate, and has higher adsorption capacity [12,13]. For this reason, a study was essentially carried out to develop an environmentally friendly adsorbent to reduce water pollution in order to preserve the water quality in the environment and guarantee the health of the population to prevent further serious destruction due to long-term exposure to this nickel (II) ion [14].

Activated carbon nanofibers (ACNFs) with abundant porosity on the surface can be used as a highly remarkable and efficient adsorption material to remove heavy metal contaminants. The highly porosity structure on ACNFs’ surface has significantly influenced the adsorption process to entrap the pollutant during water purification [15,16,17,18]. Abdullah and co-workers [19] have synthesized ACNFs composited with manganese(IV) oxide by using polyacrylonitrile (PAN) as a precursor. The authors reported that the fabrication of PAN-based ACNFs exhibited a higher adsorption capacity of 7 mg/g towards Pb(II) ions compared with commercial granular activated carbon due to high porosity structure, larger specific surface area, and the presence of numerous active functional groups on PAN-based ACNFs surface, such as oxygenated functional groups [19]. Currently, PAN is an expensive high-yielded carbon precursor because it is derived from depleted petroleum resources [20]. Subsequently, it is fundamental to foster a greener antecedent material dependent on sustainable plant-based materials as the demand for ACNFs is increasing and it is urgent to have a low-cost, sustainable, and environmentally friendly precursor [21,22]. To reduce the cost of precursor material, PAN polymer is blended with lignin in this study. Lignin is one of the renewable sources composed of large carbon composition that can widely be obtained from by-products of the pulp and paper industry [23,24]. It constitutes 25–35% of the carbon based, depending on the plant type, which with so many different plants are rich in lignin resources [25]. However, Chatterjee and Saito [26] stated that due to the fact that lignin is considered to be the least reactive substance, the solid resulting from the thermal process of lignin influences the development of the microporous nature of the final porosity on the carbon material surface. In conjunction to induce and widen the porosity structure on ACNFs’ surface, a low molecular weight of poly(ethylene glycol) (PEG) was added in this study to play the role as a porogen to develop pores on the surface of the nanofibers and to obtain a higher adsorption surface area. From their previous study, Kusumocahyo and co-workers [27] reported that the addition of PEG improved the porosity and morphology properties of poly(ethylene terephthalate) (PET) resin membranes, which has great potential for the application of water treatment processes. Therefore, the macroporosity structure and the amount of porous population on the nanofiber surface can be significantly increased by selective dissolution technique by washing out PEG from the fabricated electrospun nanofiber to obtain a higher adsorption capacity of contaminant onto the adsorbent [28,29].

In this study, the ACNFs is fabricated by using an electrospinning technique and utilized as an adsorbent for the removal of nickel(II) ions from the water system. Several adsorption parameters were thoroughly conducted, such as initial concentration, pH, and contact time, to determine the adsorption mechanism between the developed ACNFs with nickel(II) ions. To the best of our knowledge, ACNFs based on PAN incorporated with lignin and PEG have not yet been reported for adsorption of heavy metal ions from aqueous samples. The development of electrospun-based ACNFs as adsorbents can be alternative materials due to the good functionality, high porosity, large surface area, and higher degree of surface reactivity, and are suitable to be used for water remediation.

## 2. Materials and Methods

Poly(ethylene glycol)(PEG) and polyacrylonitrile(PAN) with molecular weight of 1450 and 150,000 g/mol, respectively, were utilized in this study (Sigma-Aldrich, Steinhem, Germany). Lignin kraft (3 wt%) and dimethylformamide (DMF, 99.8%), potassium hydroxide (KOH, 85%) and hydrochloric acid (HCl, 95%) (Sigma-Aldrich, Steinhem, Germany) were also used. Nickel standard solution (Ni, 1000 mg/L) was used for the sorption experiment (Scharlau, Barcelona, Spain).

### 2.1. Preparation of Electrospun PAN/Lignin/PEG Nanofibers by Using Electrospinning Technique

A polymer solution which consists of the mixture of PAN/lignin/PEG and DMF as a solvent was prepared. Weight ratio PAN/Lignin was diverse, 9:1, 8:2, and 7:3, and PEG was added variedly by 10, 20, and 30 wt% for each PAN/lignin ratio solution. The mixture of the solution was stirred until complete dissolution. The polymer solution was then filled into a 5 mL syringe attached to an 18G needle. Electrospinning parameters were set at a constant controlled pump rate of 2 mL/h and the voltage was supplied at 18 kV. An aluminum foil was used as a collector for the nanofiber mats, which was placed 10 cm from the tip of the needle. The formed nanofibers were put in a vacuum desiccator to dry before characterization.

### 2.2. Selective Dissolution Process and Preparation of Carbon Nanofibers (CNFs)

The nanofibers formed by the electrospinning process were immersed in hot deionized water of 80 °C for about one hour for selective dissolution. This process was conducted to remove PEG from the fiber structure. Subsequently, the electrospun nanofibers were dried in an oven at 60 °C for 3 h. The dried electrospun fibers were then thermally heated from room temperature to 250 °C for 3 h in air at a heating rate of 10 °C/min for stabilization. After stabilization, the nanofibers were then placed in a tube furnace and carbonized at 1000 °C for 1 h under a nitrogen atmosphere at a heating rate of 10 °C/min.

### 2.3. Preparation of Activated Carbon Nanofibers (ACNF)

A total of 1 g of carbon nanofibers was placed in a beaker containing 4 g of potassium hydroxide, KOH, and 10 g of deionized water. Then, the mixture of the solution was stirred at 60 °C for 4 h. The mixture was dried overnight in the oven at 100 °C and then heated in the furnace at 900 °C for 2 h. The heating rate in the furnace is constant 10 °C/min with a nitrogen gas flow of 200 mL/min. After thermal activation, the sample was washed with 5 wt% hydrochloric acid followed by distilled water to remove all impurities. Then, the activated carbon nanofiber was dried overnight in an oven at 100 °C.

### 2.4. Characterizations of PAN/Lignin ACNFs

ACNFs or PAN/Lignin ACNFs were characterized with several equipment and facilities. Fourier emission scanning electron microscopy (FESEM) (JEOL JSM-7600F, Tokyo, Japan) was used to determine the surface morphology of the nanofibers and the average fiber diameter was determined using ImageJ software (downloaded at https://imagej.net/Downloads (accessed on 1 September 2021)) with 200 readings per sample. Attenuated total reflectance Fourier transform infrared spectroscopy (ATR-FTIR) spectrometer (Perkin Elmer Spectrum RXI, Waltham, MA, USA) was used to determine the functional groups of the samples. Differential Scanning Calorimetry (DSC) spectrometer (Mettler Toledo Model 822, Greifensee, Switzerland) was used to determine thermal behavior of the samples. The samples were sealed within an aluminum pan and heated from 50 to 350 °C under a nitrogen atmosphere. The heating rate was set at 10 °C/min and the nitrogen flow rate of 50 mL/min.

### 2.5. Adsorption Study of Nickel(II) Ion

The adsorption of nickel(II) ion on ACNFs was investigated in the batch adsorption method. The influence of various parameters, including initial nickel(II) ion concentration, pH adjustment, and contact time, were investigated. Different concentrations of the nickel(II) ion solution (25 ppm to 100 ppm) were prepared from the appropriate nickel(II) ion stock solution with deionized water. A total of 15 mL of the nickel(II) ion solution was placed in a fixed volume conical flask. Total amounts of 50 mg ACNFs adsorbent were added to the pH 3 of nickel(II) ion solution with constant stirring at 200 rpm and shaken for 1 h to establish equilibrium. Then, the adsorbent was separated from the solution through a filter to achieve solid–liquid separation. The filtrate equilibrium concentration, C_e_, was measured using a by using inductively coupled plasma atomic emission spectroscopy (ICP-AES) (Perkin Elmer Optima 2100 DV, Shelton, CT, USA). The amount of metal adsorbed per unit mass (adsorption capacity) was calculated using the formula as shown in Equation (1):

(1) $$Q_{e}=\frac{C_{i}-C_{e}}{m}V$$

where *Q_e_* is the amount of metal ion adsorbed at equilibrium (mg.g^−1^), C_i_ and C_e_ are the initial and equilibrium concentration of metal ions in solutions (mg.L^−1^), m is the mass of the adsorbent (g), and V is the volume of nickel (II) ion solution (L).

## 3. Results and Discussion

### 3.1. Electrospun PAN/Lignin/PEG Nanofibers

**Figure 1:**
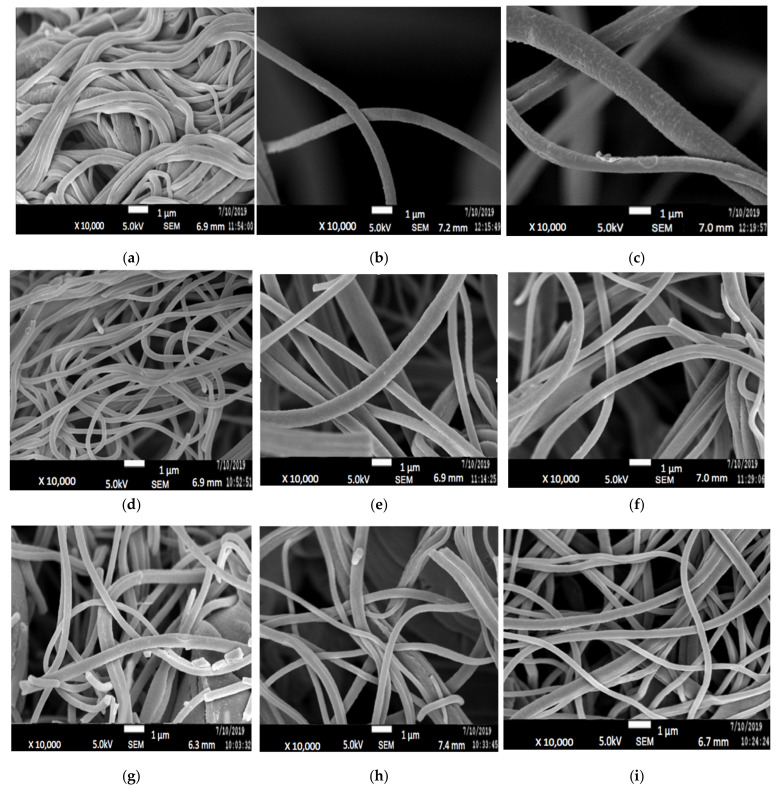
FESEM images of (**a**) 9:1 PAN/Lignin 10 wt% PEG; (**b**) 9:1 PAN/Lignin 15 wt% PEG; (**c**) 9:1 PAN/Lignin 20 wt% PEG; (**d**) 8:2 PAN/Lignin 10 wt% PEG; (**e**) 8:2 PAN/Lignin 15 wt% PEG; (**f**) 8:2 PAN/Lignin 20 wt% PEG; (**g**) 7:3 PAN/Lignin 10 wt% PEG); (**h**) 7:3 PAN/Lignin 15 wt% PEG; (**i**) 7:3 PAN/Lignin 20 wt% PEG at 10,000× magnification.

**Figure 2:**
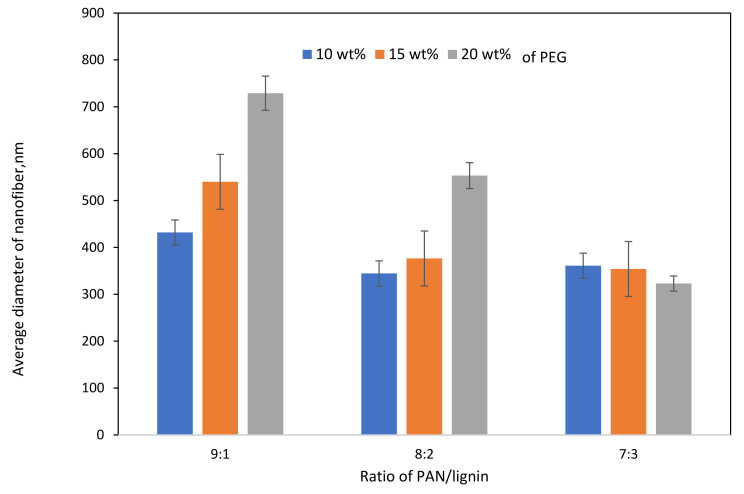
Effect of PAN/Lignin ratio and weight percent of PEG on the average diameter of nanofibers.

FESEM images of electrospun PAN/lignin nanofiber using various concentrations of PAN/lignin and weight percent of PEG are shown in Figure 1. Figure 1a–c shows the FESEM images of 9:1, Figure 1d–f are 8:2, and Figure 1g–i the ratio of 7:3 with a different weight percent of PEG; 10–20 wt% to investigate the effect of incorporating the different PAN/lignin ratio and weight percent of PEG on the average diameter of nanofiber. The average diameter of the fibers decreases slightly as the amount of added lignin increases, as illustrated in Figure 2. Nordin et al. made a similar observation concerning the decreasing diameter of fiber network in the presence of a higher content of lignin [30]. As furthering increases the amount of lignin concentration, the spinning polymer solution viscosity has been less viscous, which leads to significant improvement on morphology of the fiber network [31]. The average diameter of fibers can also be affected by the amount of PEG added. The addition of PEG to 8:2 and 9:1 PAN/lignin results in a greatly increased average diameter of the electrospun fibers. The average diameters of 8:2 PAN/lignin electrospun fibers with 10 wt%, 15 wt%, and 20 wt% PEG are 345 ± 18 nm, 377 ± 8 nm, and 553 ± 10 nm, respectively, while 9:1 PAN/lignin electrospun fibers with 10 wt%, 15 wt%, and 20 wt% PEG are 432 ± 120 nm, 540 ± 9 nm, and 729 ± 90 nm, respectively. However, the various weight percentage of PEG in 7:3 PAN/lignin, the average diameter of the fibers almost constant. From the FESEM micrographs, a significant appearance of beads and non-uniform fibers can be noticed for 7:3 PAN/lignin with 10 wt% of PEG. However, as the PEG content increased to 20 wt%, the morphology of nanofibers was uniform and had extremely neat fiber structure with extremely small fiber diameter compared with others. Therefore, a ratio of 7:3 PAN/lignin with 20 wt% PEG was selected for the synthesis of ACNFs, which has the best morphology due to the small average diameter and the absence formation of beads [30].

**Figure 3:**
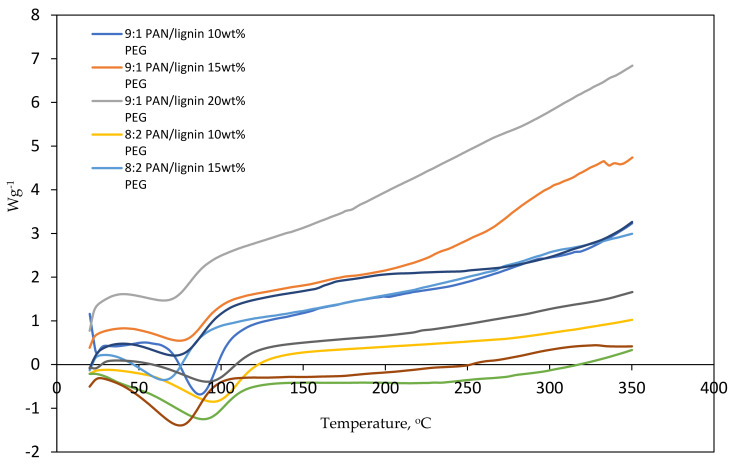
DSC thermogram of electrospun nanofibers.

Figure 3 shows the DSC thermogram of PAN/lignin/PEG nanofibers. All electrospun nanofibers with different blend ratios show a similar pattern of peaks indicating the endothermic enthalpy peak curve. The peaks indicating the glass transition temperature (T_g_) that mark the transition from glass to a rubbery state can be observed in the temperature range between 64 and 96 °C. Some of the nanofibers show a broad peak around 180 °C and 300 °C, which could represent the exothermic reaction that happened due to the cyclization of nitrile groups, dehydrogenation, and oxidation of the PAN in the nanofibers [32].

### 3.2. PAN/lignin/PEG-Based Porous Carbon Fibers

#### 3.2.1. Morphological Study of PAN/Lignin Electrospun Fibers before and after Selective Dissolution

**Figure 4:**
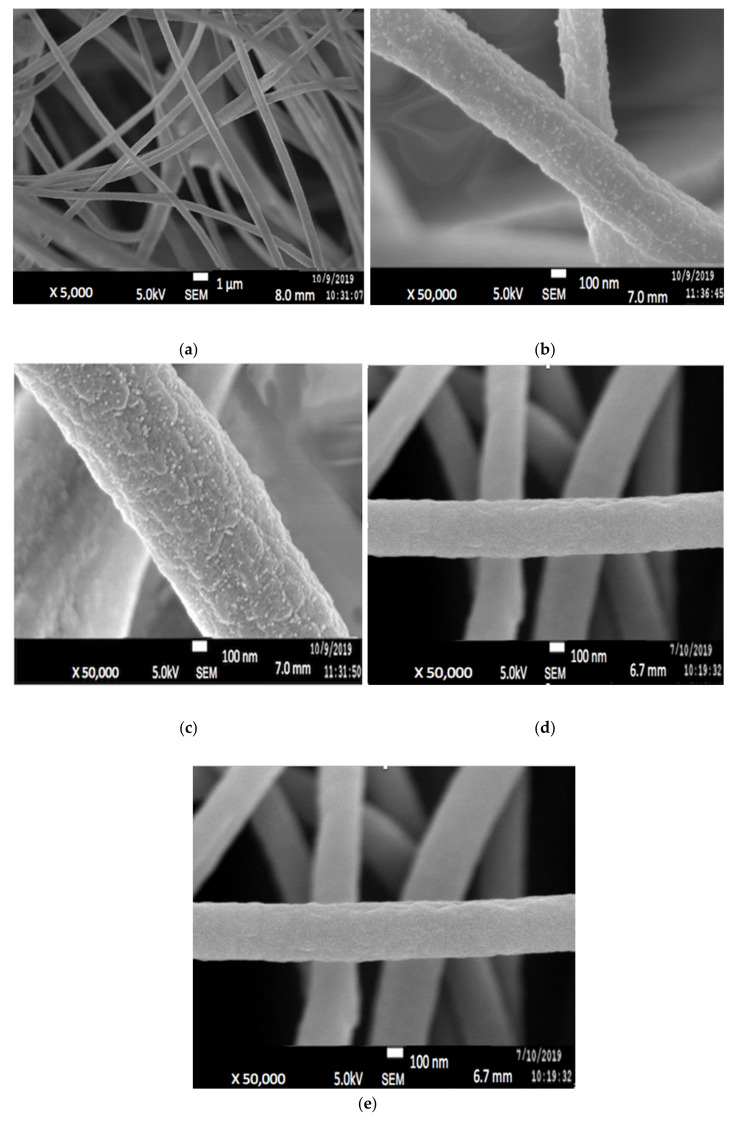
FESEM images of PAN/Lignin/PEG: (**a**) electrospun fibers; (**b**) electrospun fibers after selectively dissolution of PEG; (**c**) stabilized electrospun fibers; (**d**) carbonized CNFs; (**e**) ACNFs.

Since only 7:3 PAN/lignin with 20 wt% PEG was chosen for preparation of ACNF, it will be denoted as PAN/lignin/PEG only from here onwards. Figure 4a illustrates the morphology of the PAN/Lignin/PEG electrospun fiber, prepared by using an electrospinning technique. The uniform fiber framework has a slightly large diameter of 452 ± 113 nm, with no formation of undesired beads on the surface. Figure 4b shows FESEM image of the electrospun fibers after selective dissolution of PEG has become rougher; white and extremely small-sized beads appeared on the surface of the nanofibers, possibly as the result of selective dissolution. PEG as a pore-forming agent was removed during selective dissolution because it is a low molecular weight compound that is readily soluble in water and can promote the development of a porous structure on the surface of the fibers. The average diameter of the fiber is 323 ± 11 nm, which has slightly reduced, but is insignificant [33,34].

#### 3.2.2. PAN/Lignin/PEG Carbon Nanofibers

**Figure 5:**
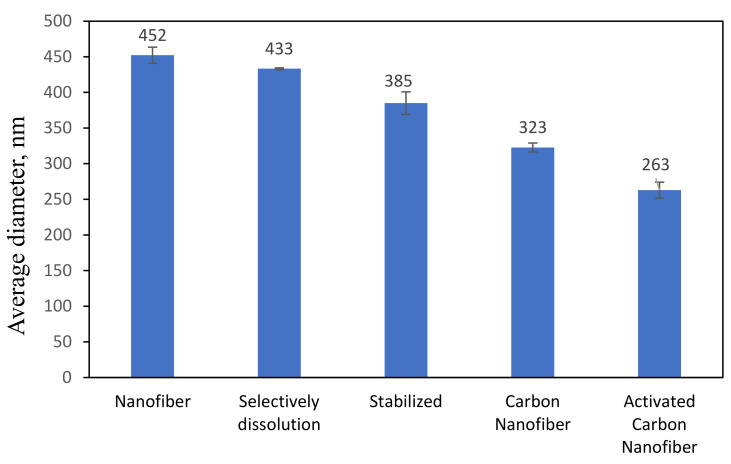
Average diameter of nanofibers.

Figure 4c shows the image of the stabilized PAN/lignin/PEG nanofibers. The appearance of the fibers changed from white to brownish yellow; however, the structure of the fibers still remained intact and no melting behavior of the fibers was observed after the stabilization process [30,35,36]. The average diameter of the stabilized nanofibers is 385 ± 15 nm (Figure 5), which was decreased compared with before stabilization, 323 ± 11 nm. The reduction in the average nanofiber diameter is due to the physical shrinkage of the nanofiber mats during heat treatment. The shrinkage of the polymer nanofiber is also affected by physical and chemical changes such as cyclization, dehydration, oxidation, and crosslinking, which can ultimately affect the diameter of the polymer [37]. After stabilization, the small beads still appear on the surface of the nanofibers, and the surface of nanofibers appears extremely rough, similar to that after selective dissolution.

Figure 4d depicts the FESEM image of PAN/lignin/PEG carbonized nanofibers. The polymer nanofiber networks are preserved, not collapsed or fused between the network after the carbonization process. The average diameter of CNFs slightly decreased to 385 ± 15 nm after stabilization and further reduced to 323 ± 6 nm after carbonization. High carbonization temperature caused the fiber of the nanofibers to shrink and the color of the fibrous mat to become black. Moreover, the increase in carbonization temperature promotes reactions such as densification of carbon and formation of higher number of pores [38]. During this heat treatment, the carbonized nanofiber is rich in carbon materials and other non-carbon materials were eliminated [39]. It is also found that the weight loss of the fibers increases with decreasing heating rate during the carbonization process [40,41]. The carbonization process starts when stabilized PAN/lignin undergoes crosslinking reaction in the non-cyclized parts of the fibers to form crosslinking and ladder structure. The reactions such as dehydrogenation and denitrogenation cause the cyclic structure to interact in the lateral direction and the non-carbon elements evaporate as a mixture of different gasses in the applied high temperature range. They develop pure CNFs with improved arrangement of the basal planes of the carbon structure due to the densification and consolidation of the carbon atoms of the nanofibers [26]. As an outcome, the decrease in fiber density, diameter, and size of nanofibers was observed due to the removal of oxygen and hydrogen atoms from the structure of low molecular weight lignin during carbonization [30]. The small beads on the surface of the nanofibers disappeared after carbonization, and the pores became more visible and produced a rougher surface of the CNFs.

#### 3.2.3. Morphology Study of PAN/Lignin/PEG Activated Carbon Nanofibers

ACNFs (Figure 4d) were found to have more porous structure compared with CNFs (Figure 4a). The average fiber diameter determined by using FESEM images of ACNFs and CNFs are 263 ± 11 nm and 323 ± 6 nm, respectively. The FESEM images clearly show that ACNFs have more compact fibers with smaller fiber diameter, while CNFs are less compact and have slightly larger fiber diameter. At the same time, several adsorbates such as ions of heavy metals, dyes, and oil can be effectively trapped more clearly on the rough surface of ACNFs [42,43]. Figure 5 shows the average diameter of PAN/Lignin nanofibers before and after the activation process. Based on the result, the average diameter of the nanofibers decreased from 452 ± 113 nm to 263 ± 11 nm after the activation process by using potassium hydroxide, KOH. This decrement of the average diameter could be explained in more detail by the reactions happening during the activation process. The activation process begins at the specific temperature for the reaction to start to occur. At heating temperature of 400 °C, some of the metallic compounds such as K, K_2_CO_3_, and emission of H_2_ gas have been produced when the carbon materials reacted with KOH. The reaction proceeded as the KOH was consumed completely at the temperature of 600 °C. Then, at temperatures higher than 700 °C, CO_2_ and K_2_O are formed due to the decomposition of K_2_CO_3_. Moreover, CO_2_ is further reduced by C in order to form CO. Additionally, metallic K has been formed due to a reduction in C on compound K_2_O and K_2_CO_3_ at higher temperatures than 700 °C [44].

The overall KOH activation mechanism shown as below:

6KOH + 2C → 2K + 3H_2_ + 2K_2_CO_3_ (2)

K_2_CO_3_ → K_2_O + CO_2_ (3)

CO_2_ + C → 2CO (4)

K_2_CO_3_ + 2C→ 2K + 3CO (5)

C + K_2_O → 2K + CO (6)

Therefore, the above activation reaction mechanisms that occur during KOH activation, as in chemical and physical activation, are essential to produce the large number of porous structures on the carbon. Additionally, the formed metallic K has the potential to intercalate and expand the carbon lattice of the carbon network. However, the expanded carbon lattice is irreversible and cannot return to a non-porous structure because intercalated K and other K compounds are removed by washing. As a result, a pore network with greater surface area, porosity and amount of surface sorption sites is formed and potentially gives higher sorption capacities to entrap the contaminants [44,45].

#### 3.2.4. Spectroscopic Study of PAN/Lignin/PEG Electrospun Fibers and CNFs

**Figure 6:**
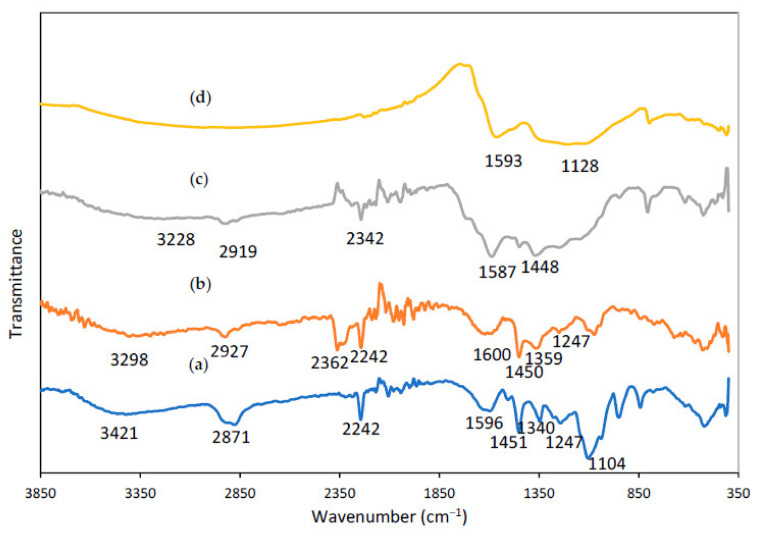
FTIR spectra of PAN/lignin/PEG nanofibers: (**a**) as-spun fiber, (**b**) after selective dissolution, (**c**) after stabilized, (**d**) CNF.

Figure 6 shows FTIR spectra of the PAN/Lignin/PEG electrospun fiber. All distinctive bands of PAN, lignin and PEG chains were observed in the expected regions of the FTIR spectra of the synthesized nanofibers with some slight shifts.

The IR spectrum of electrospun fiber (Figure 6a) describes a medium sharp peak around 3421 cm^−1^ with associated with stretching bending vibration of O-H group of lignin and PEG. The peak at 2871 cm^−1^, 1451 cm^−1^, 1340 cm^−1^ and 1247 cm^−1^ belong to the aliphatic -CH stretching groups (-CH, -CH_2_, -CH_3_) of lignin, respectively. The characteristic transmission band at 2242 cm^−1^ in the IR spectrum are assigned to the stretching and bending vibration of nitrile C≡N groups for PAN [46]. The absorption band at 1104 cm^−1^ are corresponding to C-O stretching bonds whereas C-O group related with stretching vibrations of ether groups C-O of lignin. Meanwhile, there are two absorption bands at around 1596 cm^−1^ and 1400 cm^−1^ belong to C=C and C-C stretching bond of aromatic ring that present in the lignin. Moreover, the sharp peak in the IR spectrum at 949 cm^−1^ shows the absorption band of C=C of alkene group in the lignin compound [47].

Figure 6b displays IR spectrum of electrospun fibers after selective dissolution process. A medium broad absorption peak intensity of -OH at 3421 cm^−1^ has been decreased indicates the removal of PEG successfully achieved using selective dissolution technique. A broad and strong peak for nanofiber after undergoes dissolution process is more intense rather than the peak for nanofiber before dissolution process. The presence of the absorption peak at 3298 cm^−1^ indicates the existence of the abundant alcoholic or hydroxyl groups -OH stretching of the residual content of the lignin [48]. The peak at 3231 cm^−1^ and 2927 cm^−1^ belongs to -CH stretch of methyl group of lignin and -CH stretch of PAN, respectively. The peak at 1620 cm^−1^ can be assigned to C=O bond of the aromatic ring whereas the peak at 1450 cm^−1^ shows the C-C aromatic ring stretching of lignin [49]. Meanwhile, the IR spectrum show a sharp peak at 2362 cm^−1^ and 2242 cm^−1^ that are belong to the compound which contain triple bond functional group. The peaks prove the C≡N stretching functional group of PAN still existed in the molecular structure and the wavenumber belonging to PAN is not shifted [50]. The results show that lignin and PAN were not affected by selective dissolution and remain within the nanofibers.

From the IR spectrum of stabilized nanofiber in Figure 6c, most of the strong and sharp peaks were retained after the stabilization process, but the intensity of absorption bands is slightly different from previous nanofiber. As can be observed from the graph, the absorption peak of nitrile located at 2362 cm^−1^ has been altered to be weakened significantly, whereas the absorption peak at 2242 cm^−1^ has disappeared. This is due to during stabilization process, where some reactions occurred such as dehydrogenation, cyclization, and the crosslinking process [51]. The functional group of C≡N of PAN molecules at the stabilization temperature are cyclized by heat treatment. The cyclization process of PAN converted the C≡N group into a stable ladder structure. Then, it will have conjugated to convert C≡N into C=N with an absorption band at 1587 cm^−1^. Moreover, the absorption intensity at 1450 cm^−1^ peak has been decreased to be around 1448 cm^−1^ due to the reaction of dehydrogenation. However, the absorption of a broad and strong peak can be observed at 3228 cm^−1^ and 2919 cm^−1^ due to residues of C-H alkyl of PAN and C-H methyl of lignin compound, accordingly [52,53].

Figure 6d illustrates the IR spectrum of carbonized fiber, showing that some of the functional group disappeared which shows that a successful reaction occurred during the process. The absorption peak of C≡N of PAN at 2242 cm^−1^ and 2362 cm^−1^ have completely disappeared because of the higher carbonization degree [53]. This phenomenon represents the cyclization process for the conversion of C≡N bonds into C=N and C-N bonds was successfully completed to form carbon nanofibers. The structure of the carbonized nanofiber only has carbon elements due to the fact that volatilization some of the non-carbon elements such as hydrogen and nitrogen were remarkably removed by volatile gases, for example, HCN, NH_3_, H_2_, and H_2_O [54]. A peak at the wavenumber of 1593 cm^−1^ belonged to C=C groups and a peak around 1128 cm^−1^ was assigned to the -CH deformation [55].

#### 3.2.5. Spectroscopic Study of PAN/Lignin/PEG ACNFs

**Figure 7:**
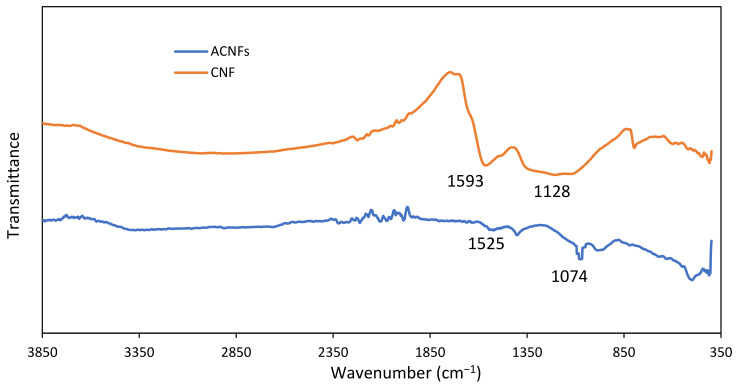
FTIR spectrum of PAN/Lignin/PEG CNF and ACNFs.

Figure 7 shows IR spectra of PAN/Lignin/PEG CNF and ACNFs. The purpose of activating carbon nanofibers by using heat and acid treatment is to increase the porous population and to generate a higher oxygen-containing surface functional group on the surface of the carbonized nanofibers [44,56]. In comparison with the CNFs spectra, the IR spectrum for ACNFs indicate the peak absorption intensity of -OH groups and C=O groups of CNFs was significantly reduced due to higher thermal treatment during chemical activation process. This indicates the successful of structural change of CNFs to form ACNFs [51]. The absorption peak at 1525 cm^−1^ corresponds to the C=C aromatic skeletal vibrations of PAN/Lignin/PEG ACNFs [30]. However, the addition of the strong and sharp absorption peak at 1074 cm^−1^ was assigned to C-O group deformation for ACNFs and can be observed due to oxygen exchange reaction after KOH activation treatment. The sharp peak indicates the increase in oxygen-containing functional group because the carbon atom on the nanofibers’ surface has been successfully oxidized to CO when it interacts with KOH [57].

### 3.3. Adsorption Study of ACNFs towards Ni(II) Ions

#### 3.3.1. Effect of Initial Concentration of Ni(II) Ions

**Figure 8:**
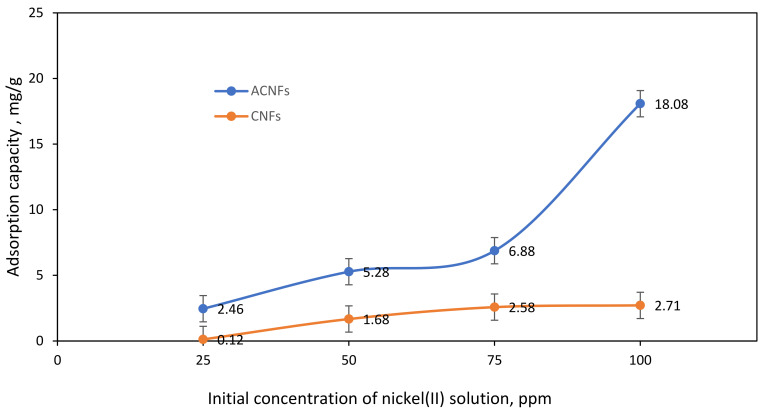
Effect of initial concentration on adsorption capacity of Ni(II) ions for ACNFs and CNFs. Condition: Solution pH = 3, Adsorbent dosage = 50 mg, contact time = 60 min.

The effect of initial concentration of Ni(II) ions within the range of 25–100 ppm onto the CNF and ACNFs were studied to evaluate the adsorption performance for the both adsorbents. Figure 8 shows the adsorption capacity of ACNFs and CNFs at different concentrations of Ni(II) solution. From the graph, the initial concentration of Ni(II) ions solution has slightly effected the adsorption capacity of nanofibers towards Ni(II) ions solution. The uptake of Ni(II) ions on CNFs gradually increases from 25 ppm to 50 ppm with the maximum adsorption capacity of 0.12 mg/g to 2.71 mg/g, respectively. When the concentration of Ni(II) ions solution is reached more than 75 ppm, the adsorption capacity of CNFs is decreased. This might be due to the limitation of the availability of active sites on the surface of CNFs [58]. In contrast, higher uptake of Ni(II) ions was observed on ACNFs with the maximum adsorption capacity of 18.08 mg/g. The adsorption capacity of Ni(II) ions has been influenced by the presence of the oxygenated surface group generated on PAN/lignin/PEG ACNFs, as evidenced by FTIR analysis. The attachment of surface oxygen groups decreases the hydrophobicity of the adsorbent surface, thus improving the interaction with Ni(II) ions and promoting the Ni(II) ions adsorption on the PAN/lignin/PEG ACNFs [30]. The adsorption capacity is rapidly increased from 25–100 ppm Ni(II) ions solution. The adsorption capacity of ACNFs at 100 ppm is 18.08 mg/g, while CNFs has only 2.71 mg/g. This finding shows that ACNFs has higher ability to adsorb Ni(II) ions compared with CNFs due to the ratio of availability of adsorption sites on ACNF surface. Other than that, high surface area of ACNF provides more penetration sites for the Ni(II) ions for the adsorption process. Therefore, 100 ppm was used as an initial concentration of Ni(II) ions solution for further adsorption study.

#### 3.3.2. Effect of pH

**Figure 9:**
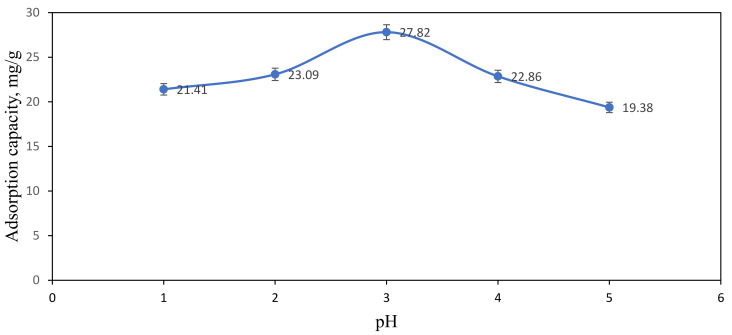
Effect of pH on adsorption capacity of Ni(II) ions. Condition: [Ni(II)] = 100 ppm, dosage of adsorbent = 50 mg, contact time = 60 min.

The removal of Ni(II) ions from aqueous solution by using ACNFs was affected by the pH of the sample solution. The pH value was chosen in the range of 1–5 to investigate the adsorption capacity of ACNFs towards Ni(II) ions. Figure 9 shows that the absorption capacity of Ni(II) ions is strongly dependent on the pH of Ni(II) ions solution. At pH 1 to 3, the absorption capacity is slightly increased from 21.41 to 27.82 mg/g. The adsorption capacity is decreased to 22.86 mg/g and 19.38 mg/g when the pH of sample solution is 4 and 5, respectively. In the highly acidic state of Ni(II) ions solution, the concentration and mobility of hydrogen ions are relatively higher because the negative functional groups located on the active site of ACNFs can bind easily with the hydrogen ions compared with Ni(II) ions [49]. This is due to the electron repulsion and competition between Ni(II) ions and hydrogen ions in binding at the surface of ACNFs that can inhibit the uptake of the metal ions such as Ni(II) ions into the active site of ACNFs [33,59]. The adsorption sites on the ACNFs for the uptake of metal ions increased with increasing pH [32]. This is due to the fact that the number of negatively charged adsorption sites increases up to pH 7 [60]. However, the decreasing adsorption capacity at pH 4 and 5 may be due to the formation of precipitates or insoluble Ni(II) hydroxides, Ni(OH)_2_ from Ni(II) cation. Due to that, adsorption capacity is not applicable at pH higher than 6 due to the higher saturation of hydroxide ions which may react with Ni(II) ions to form Ni(OH)_2_ precipitates [61,62,63]. Therefore, the adsorption capacity study is not tested at pH higher than 6 and pH 3 was used for further adsorption study.

#### 3.3.3. Effect of Contact Time

**Figure 10:**
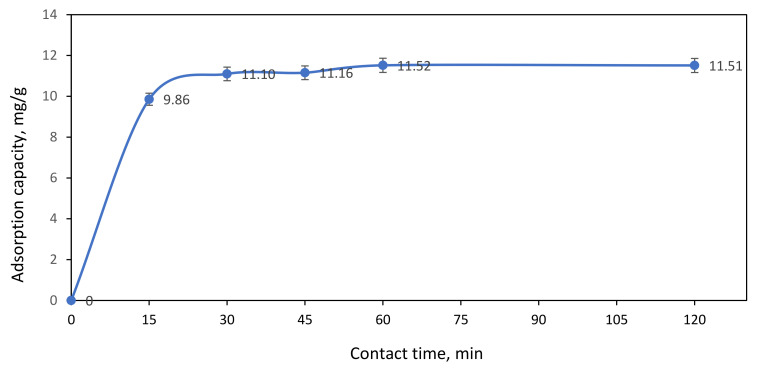
Effect of contact time on adsorption capacity of Ni(II) ions. Condition: [Ni(II)] = 100 ppm, dosage of adsorbent = 50 mg, pH = 3.

Figure 10 demonstrated the effect of contact time on the adsorption of 100 ppm Ni(II) ions by ACNFs. From the graph, it shows that the adsorption capacity gradually increases from 9.86 to 11.52 mg/g with the adsorption time until 60 min. This might be due to the high availability of free adsorption sites and larger surface area of ACNFs [30]. However, as the contact time progresses from min, the adsorption sites of ACNFs are saturated. Thus, the optimum of adsorption capacity on ACNFs is at 60 min as the graph become plateau from 60 to 120 min. Therefore, this phenomenon indicates that equilibrium adsorption between ACNFs and Ni(II) ions has been successfully achieved when the adsorption time is 60 min.

## 4. Conclusions

PAN/Lignin/PEG-based porous ACNFs was successfully synthesized by electrospinning technique with the best characteristics in terms of morphology, smallest diameter, no bead formation, and more uniform fiber diameter. PAN/Lignin/PEG were mixed and used as carbon precursor to obtain carbon nanofiber. Incorporation of lignin in the fibers affected the diameter of the fibers produced to some extent. PEG role as a porogen through selective dissolution techniques is successful, which can be seen from the FESEM images of the fibers becoming rougher and the presence of extremely small beads on the surface of the fibers after selective dissolution process. The application of PAN/lignin-based highly porous ACNFs as adsorbents for nickel ions was successfully investigated. The effect of initial concentration, the effect of contact time, and the effect of pH on the adsorption capacity of ACNFs were examined. The results show the adsorption capacity of ACNFs at 100 ppm Ni(II) ions solution gave the highest percentage removal of 60%, with adsorption capacity of 18.09 mg/g compared with CNFs, which had adsorb only at 2.7 mg/g. For the pH parameter, the adsorption capacity of ACNFs at pH 3 is 27.82 mg/g showed the maximum amount adsorbed than other pH values. Thus, the optimum pH of adsorption capacity is at pH 3. Meanwhile, the effect of contact time from 15 min contact with Ni(II) ions solution to 120 min was investigated. The outcome revealed that the optimal adsorption capacities of 11.52 mg/g by ACNFs was achieved at 60 min contact time between ACNFs and Ni(II) ion solution. At this time, the adsorption equilibrium was reached. The study demonstrates the fabrication of ACNFs from PAN/lignin/PEG electrospun nanofibers have potential as adsorbents for the removal of heavy metal contaminants.

## Data Availability

The data presented in this study are available on request from the corresponding author.
